# The Systematics and Evolution of Gymnosperms with an Emphasis on a Few Problematic Taxa

**DOI:** 10.3390/plants13162196

**Published:** 2024-08-08

**Authors:** Yong Yang, Zhi Yang, David Kay Ferguson

**Affiliations:** 1 Co-Innovation Center for Sustainable Forestry in Southern China, State Key Laboratory of Tree Genetics and Breeding, College of Life Sciences, Nanjing Forestry University, Nanjing 210037, China; 2 Department of Paleontology, University of Vienna, 1010 Vienna, Austria

**Keywords:** gymnosperms, phylogenomics, character evolution, systematics

## Abstract

Gymnosperms originated in the Middle Devonian and have experienced a long evolutionary history with pulses of speciation and extinction, which resulted in the four morphologically distinct extant groups, i.e., cycads, *Ginkgo*, conifers and gnetophytes. For over a century, the systematic relationships within the extant gymnosperms have been debated because different authors emphasized different characters. Recent phylogenomic studies of gymnosperms have given a consistent topology, which aligns well with extant gymnosperms classified into three classes, five subclasses, eight orders, and 13 families. Here, we review the historical opinions of systematics of gymnosperms with special reference to several problematic taxa and reconsider the evolution of some key morphological characters previously emphasized by taxonomists within a phylogenomic context. We conclude that (1) cycads contain two families, i.e., the Cycadaceae and the Zamiaceae; (2) *Ginkgo* is sister to cycads but not to conifers, with the similarities between *Ginkgo* and conifers being the result of parallel evolution including a monopodial growth pattern, pycnoxylic wood in long shoots, and the compound female cones, and the reproductive similarities between *Ginkgo* and cycads are either synapomorphic or plesiomorphic, e.g., the boat-shaped pollen, the branched pollen tube, and the flagellate sperms; (3) conifers are paraphyletic with gnetophytes nested within them, thus gnetophytes are derived conifers, and our newly delimited coniferophytes are equivalent to the Pinopsida and include three subclasses, i.e., Pinidae, Gnetidae, and Cupressidae; (4) fleshy cones of conifers originated multiple times, the Podocarpaceae are sister to the Araucariaceae, the Cephalotaxaceae and the Taxaceae comprise a small clade, which is sister to the Cupressaceae; (5) the Cephalotaxaceae are distinct from the Taxaceae, because the former family possesses typical female cones and the fleshy part of the seed is derived from the fleshiness of integument, while the latter family has reduced female cones and preserves no traces of the seed scale complexes.

## 1. Introduction

Gymnosperms are a group of seed plants with unisexual reproductive organs (except for the gnetophytes) and naked ovules and seeds (partially enveloped in the gnetophtyes) [1]. Unlike flowering plants, they lack vessels in secondary xylem (apart from the gnetophytes), lack flowers and fruits, and lack double fertilization (except for the gnetophytes) and endosperm normally developed from the haploid gametophytes [1]. The fundamental difference between gymnosperms and angiosperms is the enclosure of ovules and seeds, naked or partiallsy naked in gymnosperms, but, in angiosperms, they are enclosed in an ovary [1].

**Figure 1:**
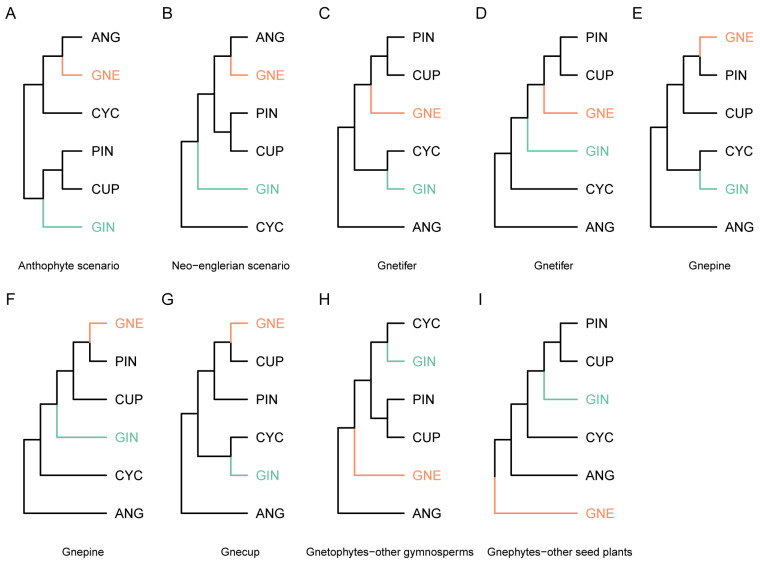
Different hypotheses on phylogenetic relationships of seed plant groups. (**A**) anthophyte scenario indicating that gnetophytes and angiosperms comprise a clade sister to cycads; (**B**) neo-englerian scenario suggesting that gnetophytes and angiosperms comprise a clade sister to conifers; (**C**,**D**), gnetifer hypothesis implying that gnetophytes are sister to conifers, (**C**) differs from (**D**) in the relationship of *Ginkgo*; (**E**,**F**), gnepine hypothesis suggesting that conifers are paraphyletic with gnetophytes sister to the Pinaceae, (**E**) differs from (**F**) in the position of *Ginkgo*; (**G**), gnecup hypothesis indicating that conifers are paraphyletic with gnetophytes sister to cupressophytes; (**H**), gnetophytes-other gymnosperms hypothesis suggesting that gymnosperms constitute a monophyletic group and gnetophytes are sister to a clade including the rest gymnosperm groups; (**I**), gnetophytes-other seed plants hypothesis denoting that gymnosperms are paraphyletic and gnetophytes are sister to a clade encompassing other seed plant groups. Colored branches and abbreviations display the jumping groups of seed plants. Abbreviations: ANG: angiosperms; CYC: cycadophytes; GIN: *Ginkgo*; GNE: gnetophtyes; PIN: Pinaceae; CUP: cupressophytes.

Gymnosperms originated in the mid-Devonian (~385 mya) [2] and have experienced a long evolutionary history with many extinctions [3], which led to the huge gaps among the four morphologically distinct extant groups, i.e., cycads, *Ginkgo*, conifers and gnetophytes. Robert Brown discovered and circumscribed gymnosperms in 1827 after he examined the enclosure of ovules and seeds in seed plants, and he found that ovules of seed plants include two types: one type is naked, while the other type is enclosed in an ovary [1,4,5]. Subsequently, the systematics of gymnosperms have drawn the attention of numerous botanists, especially the phylogenetic relationships of a few problematic groups (Figure 1) [1]. The main debates on gymnosperm systematics include the following: (1) Is *Ginkgo* sister to cycads or to conifers? (2) Do conifers constitute a monophyletic group or not? (3) How to classify those conifer families lacking typical female cones (taxads)? (4) What are the systematic relationships of the gnetophytes?

Molecular phylogenetic studies are based on DNA sequences, and the techniques for obtaining sequences have developed rapidly from the Sanger Sequencing to the Next-generation Sequencing. The molecular phylogenies of the Sanger Sequencing phase involve one or a few DNA markers/fragments and give rise to generally poorly resolved or even conflicting phylogenetic trees, while those of the Next-generation Sequencing phase employ complete plastomes and/or a large number of nuclear genes, and usually give rise to well-resolved phylogenomic trees. This is particularly true for gymnosperm systematics. Early molecular phylogenetic studies based on one or a few DNA fragments gave conflicting topologies of phylogeny of extant gymnosperms, e.g., the phylogenetic position of *Ginkgo* and gnetophytes [1,6]. However, recent phylogenomic studies based on a large number of nuclear genes have yielded consistent relationships [7,8,9,10]. According to these phylogenomic results, living gymnosperms contain two major clades, one clade including cycads (cycadophytes) and *Ginkgo* (ginkgophytes) and the other clade consisting of conifers and gnetophytes; conifers are paraphyletic with the Pinaceae sister to gnetophytes, and the remaining conifer families belong to the cupressophytes. Yang et al. [10] integrated the phylogenomic results and morphological characters, and classified the living gymnosperms into three classes (Cycadopsida, Ginkgoopsida, and Pinopsida), five subclasses (Cycadidae, Ginkgoidae, Cupressidae, Pinidae, and Gnetidae), eight orders (Cycadales, Ginkgoales, Araucariales, Cupressales, Pinales, Ephedrales, Gnetales, and Welwitschiales), and 13 families (Cycadaceae, Zamiaceae, Ginkgoaceae, Araucariaceae, Podocarpaceae, Sciadopityaceae, Cupressaceae, Cephalotaxaceae, Taxaceae, Ephedraceae, Gnetaceae, and Welwitschiaceae) (Table 1).

Due to these molecular phylogenetic/phylogenomic advances, the controversial questions of gymnosperms have been elucidated during the last three decades. In this paper, we review the advances of molecular systematics of gymnosperms with special reference to the problematic taxa. Our main purposes are to (1) comb through the historical opinions on the systematic relationships of these problematic groups; (2) demonstrate how the molecular systematic studies have challenged those historical hypotheses; and (3) elucidate the evolution of the taxonomic characters according to the recent phylogenomic results.

## 2. Phylogeny and Taxonomy of Problematic Taxa

### 2.1. Cycads

The cycadophyte lineage originated very early in the Pennsylvanian [11] and has experienced fluctuations of diversifications and extinctions [12]. The long and complicated evolutionary process has given rise to the modern 10 genera and ~380 species that are distributed in the tropics of Africa, Asia, Australia and the Americas.

Cycads are palm-like; some have above-ground trunks unbranched or bifurcately branched, whereas others possess no above-ground trunks. Stems are manoxylic with massive pith and cortex. The leaves include two types: cataphylls are scale-like and spirally arranged; and trophophylls are compound and large, with few-to-many leaves clustered at the trunk apex. The leaf traces are girdling, viz. originate from the stele and extend horizontally before entering the leaf petiole. All cycads are dioecious. Male cones are fusiform-cylindric, normally one at the top of the trunk amidst the compound leaves and bear numerous microsporophylls spirally arranged along the cone axis. The pollen is boat-shaped. Female cones are globose and at the top of the trunk, possessing numerous megasporophylls. These megasporophylls are pinnatifid (Cycadaceae) or peltate (Zamiaceae). Each megasporophyll bears two or several colorful seeds proximally. Seeds are subglobose or subcylindrical; the seed coat is differentiated into three layers, i.e., a fleshy sarcotesta (usually colorful, pink, orange or red-brown), a woody sclerotesta and a membranous endotesta. Pollen tubes of cycads are branched, developing as haustoria to obtain nutrition from the female gametophyte. The sperm is flagellate, which leads to the zoidogamy in cycads.

Cycads represent the earliest diverged lineage among the living gymnosperms. They possess a number of characters that are similar to monilophytes and seed ferns. Stems are unbranched or bifurcately branched. The compound leaves are circinate when young; like *Ginkgo*, cycads have flagellate sperms [13]. Recent phylogenomic studies have consistently shown that cycads and *Ginkgo* form a clade that is sister to other living gymnosperms [8,9].

There were different opinions on how to classify the living cycads at the family level. Pilger [14] divided cycads into two families, i.e., the Cycadaceae and the Zamiaceae. Johnson [15] established the Stangeriaceae. Stevenson [16] erected the Boweniaceae. Kubitzki [17] accepted all these families and classified cycads into four families, i.e., the Cycadaceae (*Cycas*), the Boweniaceae (*Bowenia*), the Stangeriaceae (*Stangeria*) and the Zamiaceae (the remaining genera). Stevenson [18] accepted three families, i.e., the Cycadaceae (*Cycas*), the Stangeriaceae (including *Stangeria* and *Bowenia*) and the Zamiaceae (the remaining genera). Molecular phylogenetic/phylogenomic studies have consistently suggested that the living cycads should be classified into two families, the genus *Cycas* alone in the Cycadaceae, and the remaining nine genera in the Zamiaceae; both the Boweniaceae and the Stangeriaceae are nested within the Zamiaceae [9,10,19,20], and thus the two families should be incorporated within the Zamiaceae.

### 2.2. Ginkgo

The ginkgophyte lineage was once diversified and widely distributed in the geological past, but currently consists of only one living species, i.e., *Ginkgo biloba* [21,22]. This living species is relictual and native to the SW, S and E of China [23]. *Ginkgo biloba* is a deciduous and dioecious tree and has unique fan-shaped leaves. There are two types of shoots, with leaves spirally arranged along the long shoot and clustered at the top of the short shoot. The short shoot has a reproductive function and possesses numerous spirally arranged scale leaves proximally and several vegetative leaves clustered distally, and normally, there are several pedunculate male cones or female cones inserted in the axils of the scale leaves at the apex of the short shoot. The male cone is pedunculate and bears many spirally arranged microsporophylls, each microsporophyll possesses two microsporangia. The pollen is boat-shaped [17]. The female cones possess a long peduncle terminated by normally two ovules or seeds. At the base of the ovule, there is an annular swollen structure called a collar. The ovule is fertilized and develops into a subglobose seed with the integument differentiated into three layers, i.e., an outer fleshy layer (sarcotesta), a middle hard layer (sclerotesta) and an inner membranous layer (endotesta). Within the endotesta, the embryo is surrounded by the green or yellowish green gametophyte.

Earlier botanists classified *Ginkgo* with the taxads because its ovulate organs (the one or two fleshy seeds terminal to a peduncle) are superficially similar to the reduced fleshy female organs of taxads [24,25]. In 1896, Japanese botanists reported the occurrence of flagellate sperms in cycads and *Ginkgo* [26,27,28,29]. Soon, taxonomists treated the ginkgophyte as a separate lineage intermediate between cycads and conifers, and some considered that *Ginkgo* is close to conifers and should belong to the coniferophytes because of its well-developed monopodial trunk and branching pattern (vs. unbranched or bifurcately branched trunk in cycads) and the pycnoxylic wood (vs. manoxylic wood in cycads) [13,17,30].

Some others, however, argued that *Ginkgo* is sister to the cycads because of their reproductive similarities including the boat-shaped pollen (vs. globose or ephedroid in conifers and gnetophytes), the branched pollen tube (usually unbranched in conifers and gnetophytes), the flagellate and motile sperms (vs. nonflagellate sperms in conifers and gnetophytes), the seed possessing a three-layered seed coat, i.e., the outermost sarcotesta, the middle sclerotesta, and the inner endotesta [31,32,33]. Fujii [34] considered that *Ginkgo* is closest to the cycads. He studied the metamorphoses of ovuliferous organs of *Ginkgo* and believed that the collar below the ovule of *Ginkgo* is a reduced sporophyll, and the stalked ovuliferous organ is a reproductive shoot bearing two sporophylls; the presence of megasporophylls in *Ginkgo* is similar to cycads. He also compared the short shoot of *Ginkgo* with densely arranged scale leaves to the stem of cycads with dense cataphylls. Moreover, he considered the homology of some other shared characters between *Ginkgo* and cycads, e.g., the spermatozoids and drupaceous seeds.

Early molecular phylogenetic studies based on one or a few barcoding markers gave rise to inconsistent results, some indicated a sister relationship between cycads and *Ginkgo* [35,36], while others suggested that *Ginkgo* is sister to conifers [37,38,39]. Recent phylogenomic studies based on plastomes and a large number of nuclear genes have consistently suggested that *Ginkgo* is sister to cycads [7,8,9,40,41]. This result implies that the similarities between *Ginkgo* and conifers are not synapomorphic but the result of parallel evolution, e.g., the branching pattern of trunk, the pycnoxylic wood and the proto-seed scale complexes.

### 2.3. Conifers

Conifers are the core gymnosperms since the species represent 58.5% of all species of living gymnosperms. Conifers are trees or shrubs, and dominant in ca. 39% forested areas on Earth, especially in the boreal forests and subtropical mountainous forests at higher elevations [1]. Conifers possess diverse leaves, needle-like in *Picea*, *Pinus* and *Cedrus*, linear in *Abies*, *Metasequoia*, *Sequoia*, *Larix*, *Pseudolarix*, *Wollemia* and *Taxus*, lanceolate in *Cunninghamia* and *Podocarpus*, elliptic in *Nageia* and *Agathis*, subulate in *Taiwania* and *Cryptomeria*, acicular in *Juniperus*, phyllode in *Phyllocladus* and scale-like in *Platycladus* and *Sabina*. Those species with typical female cones are usually monoecious, while those species with fleshy cones are normally dioecious. Male cones are simple and consists of a central axis bearing many microsporophylls in Cupressaceae, Araucariaceae, Pinaceae and Podocarpaceae, or are compound in taxads; the male cones are singular or clustered. Conifers usually possess female reproductive organs consisting of a central axis bearing a variable number of seed scale complexes. The Taxaceae are exceptional, because there is no evidence for seed scale complexes. Conifers are widely distributed throughout the globe, from north to south and from west to east, but they are not evenly distributed on Earth, some places have very high species richness, e.g., Himalayas, Borneo to New Caledonia and western North America [1,42].

Conifers have been considered as a monophyletic group for a long time. Whether conifers constitute a monophyletic group or not largely depends on the phylogenetic position of the gnetophytes and the derivation of the reduced female cones in taxads. Based on one or a few fragments of barcoding markers, some early molecular phylogenetic studies suggested that conifers constitute a monophyletic group [39,43], whereas some other studies suggested that conifers are paraphyletic with gnetophytes nested within conifers [36,37,38]. Recent phylogenomic studies have established that conifers are paraphyletic with gnetophytes sister to the Pinaceae [7,8,9]. Some early authors included ginkgophytes within the coniferophytes [13,30]; here, we consider that coniferphytes contain only conifers and gnetophytes, but exclude *Ginkgo* in accordance with the recent phylogenomics of gymnosperms.

Most taxonomists employing morphological characters classified conifers into two groups, one group possessing typical female cones, e.g., the Pinaceae, the Araucariaceae and the Cupressaceae, with the other group having atypical female cones (usually reduced and fleshy), e.g., the Podocarpaceae, the Cephalotaxaceae and the Taxaceae [44]. Florin [45] thought that the unusual female cone of the Taxaceae may have independently originated from the female reproductive organs of *Lebachia* and was not reduced from a compound cone like other conifers; thus, the family merited a higher taxonomic rank, viz. Taxopsida. Cheng and Fu [46] disagreed with Florin’s opinion and classified conifers into Pinales, Podocarpales, Cephalotaxales and Taxales, which suggests that female cones of conifers reduced gradually from the typical female cones of the Araucariaceae, the Pinaceae, the Taxodiaceae and the Cupressaceae to the uni-ovulate organs of the Taxaceae via the transitional cones of the Podocarpaceae and the Cephalotaxaceae. Molecular phylogenetic/phylogenomic studies, however, did not validate the hypotheses of Florin [45] and Cheng and Fu [46], but have suggested that the reduced and fleshy female cones originated independently in the Podocarpaceae, the Cephalotaxaceae and the Taxaceae [7,8,47]. According to recent phylogenomic studies, conifers should be classified into three subgroups: the first subgroup containing only the Pinaceae (Pinales), the second subgroup encompassing two southern conifer families, i.e., Araucariaceae and Podocarpaceae (Araucariales), and the third subgroup including the Sciadopityaceae, the Cupressaceae, the Cephalotaxaceae and the Taxaceae (Cupressales); the family Pinaceae alone constitutes Pinidae, and the remaining conifer families make up the Cupressidae [10]. Farjon [42] also accepted a seven-family classification of conifers.

The Podocarpaceae are distributed mainly in the Southern Hemisphere, and there were different opinions regarding the taxonomy: one family or multiple families. Keng [44] contended that *Phyllocladus* should be classified as a separate family, viz. the Phyllocladaceae. Kubitzki [17], Bobrov et al. [48], and Farjon [49] followed this treatment. Fu [50] noticed that *Nageia* differs from other genera of the Podocarpaceae in the broad leaves having multiple parallel veins but no midvein and believed that there was a multi-nerved evolutionary line including Cordaitales, the Araucariaceae, the Nageiaceae and gnetophytes; thus, he classified *Nageia* in a separate family, the Nageiaceae. Doweld even went so far as to divide the Podocarpaceae into 12 families [3]. Molecular phylogenomic studies have consistently suggested that these small families are nested within the Podocarpaceae [51] and thus should be included in the Podocarpaceae [10].

The taxonomy of *Sciadopitys*, Taxodiaceous and Cupressaceous plants has been controversial for a long time [52,53,54,55]. Many studies based on morphological characters divided them into two families, i.e., Taxodiaceae bearing female cones with spirally arranged seed scale complexes and Cupressaceae having female cones with opposite or ternately whorled seed scale complexes [14,46]. The discovery of *Metasequoia* challenged this traditional opinion; *Metasequoia* shows close affinity to *Sequoia* and *Sequoiadendron*, but possesses opposite phyllotaxy (including leaf position and seed scale complex position), which is similar to the Cupressaceae s.s., suggesting the incorporation of the Taxodiaceae and the Cupressaceae [53,54,55,56]. Eckenwalder [53] treated *Sciadopitys* as a subfamily of the Cupressaceae s.l. Schlarbaum and Tsuchiya [57] found that *Sciadopitys* has an unusual base number of chromosomes (x = 10), different from the base number of Taxodiaceae (x = 11), and thus argued that *Sciadopitys* should be treated as a separate family, the Sciadopityaceae. Molecular phylogenetic/phylogenomic studies have consistently suggested that the Taxodiaceae s.l. are polyphyletic, *Sciadopitys* diverged first, and the remaining taxodiaceous genera plus the Cupressaceae s.s. constitute a monophyletic group (the Cupressaceae s.l.), which is sister to a small clade including the Cephalotaxaceae and the Taxaceae [7,8,39]. Based on these studies, *Sciadopitys* alone should be classified in a separate family, i.e., the Sciadopityaceae, while the Taxodiaceae should be incorporated into the Cupressaceae [10].

The relationships of the Cephalotaxaceae and the Taxaceae have been controversial for a long time. Some researchers considered that the Cephalotaxaceae should be separated from the Taxaceae and even upgraded to an order because the Cephalotaxaceae bear typical female cones, with the cone axis bearing a few pairs of bracts each having two axillary orthotropous ovules [46,58]. Some others believed that the Cephalotaxaceae should be included in the Taxaceae [59,60]. Molecular phylogenies based on one or a few DNA markers gave rise to different relationships; some suggested that *Cephalotaxus* is sister to the Taxaceae [61,62], so it was reasonable to classify them as two separate families, viz. the Cephalotaxaceae and the Taxaceae [63], while others indicated that *Cephalotaxus* is nested within the Taxaceae, and thus they should be merged into a single family, viz. the Taxaceae [64,65]. Recent phylogenomic studies, however, consistently suggested that *Cephalotaxus* is sister to the Taxaceae. Based on multidisciplinary evidence from morphology, embryology, chemistry and phylogenomics, Yang et al. [58] concluded that the Cephalotaxaceae should be separated from the Taxaceae.

### 2.4. Gnetophytes

The gnetophyte lineage belongs to the coniferophytes since recent phylogenomics studies consistently suggest a gnepine relationship [7,8,9]. This lineage includes three morphologically distinct genera, i.e., *Ephedra*, *Gnetum* and *Welwitschia* [66,67]. Species of *Ephedra* are shrubs, subshrubs or rarely small trees, possessing articulate branches with longitudinally striated internodes and swollen nodes. Leaves are extremely reduced, membranous, two or three free or fused into a sheath enclosing the node. It is not the reduced leaves but the young and green twigs that carry out photosynthesis. The male cones are compound and possess a number of paired or ternately whorled bracts; with the exception of the proximal pair, each bract subtends a pair of dorso-ventral positioned bracteoles that are basally fused and enclose a central microsporangiophore; three to eight microsporangia are terminal to a microsporangiophore. The female cones are reduced and possess two to 13 pairs/whorls of bracts with the uppermost pair/whorl enclosing 1–3 chlamydosperms. *Gnetum* species are ligneous lianas, rarely trees that are evergreen. The leaves are opposite, petiolate, broad with pinnate-venation. Species of *Gnetum* are dioecious or functionally dioecious. The male spikes are dichasially branched, the bracts are reduced and annular, normally each annular bract subtending several whorls of male units. The male unit consists of a pair of bracteoles enclosing a central microsporangiophore. Each microsporangiophore is terminated by two microsporangia. There is normally one whorl of chlamydosperms above the male unit whorls. The female spikes are dichasially branched, and have a number of annular bracts. The annular bract subtends a whorl of chlamydosperms. Each chlamydosperm possesses two outer envelopes with an apical opening through which a micropylar tube is elongated. *Welwitschia* is a dwarf shrub and possesses a pair of huge strap-shaped leaves with numerous parallel veins. This genus is functionally dioecious, and either male cones or female cones arise in the central area between the two leaves. The male cones are compound, and possess many pairs of bracts, each bract subtends a ‘bisexual’ unit with six microsporangiophores basally fused and enclosing a central aborted ovule. The female cones are dry at maturity, and possess numerous bracts, each bract subtends a chlamydosperm, which is bilateral and has two outer envelopes enclosing an ovule with an elongated micropylar tube. The three living genera of gnetophytes occupy distinctive habitats. Species of *Ephedra* are adapted to dry and cold conditions, those of *Gnetum* favor humid and warm places, and the single species of *Welwitschia* lives in dry and warm coastal habitats in southwestern Africa [66]. All gnetophytes (or chlamydospermophytes) have ovules and seeds partially enclosed in one or two layers of envelopes with the micropylar tube exposed; the envelope of gnetophytes originated from the fusion of two foliar organs [68].

Gnetophytes possess a combination of unusual characters. Vessels are present in the secondary wood of all the living genera [33,69]. Broad-leaves of *Gnetum* with pinnate venation are similar to the leaves of dicots of angiosperms. *Ephedra* usually possesses normally unisexual cones, but occasionally bisexual cones, with the uppermost pair enclosing two chlamydosperms and the lower pairs subtending microsporangiophores in *Ephedra campylopoda* (= *E. foeminea*); in *Gnetum*, most species have male cones with one whorl of aborted chlamydosperms above the microsphorangiophores [66,67,70]; in *Welwitschia*, the male cone consists of a bisexual reproductive unit with one aborted ovule surrounded by six basally fused microsphorangiophores [67]. These bisexual cones are different from the unisexual cones of other gymnosperms, but similar to the bisexual flowers of angiosperms. Archegonia are present in *Ephedra* but absent in *Gnetum* and *Welwitschia* [66]. Double fertilization was observed in all three extant genera, where one male gamete is fused to the egg and the other male gamete is fused to the ventral nucleus or a free nucleus, but unlike angiosperms, only one zygote developed while the other aborted [71].

Gnetophytes are the most controversial group among extant gymnosperms. Some authors considered that the gnetophytes are monophyletic [33], some thought that gnetophytes are paraphyletic [72], and some others even suggested that the gnetophytes are polyphyletic [73,74,75,76,77,78]. Molecular phylogenetic/phylogenomic studies have consistently suggested that the gnetophytes are monophyletic [7,8]. As for the phylogenetic relationships of the gnetophytes, cladistic analyses based on morphological characters suggested the anthophyte hypothesis [76,77,78] or neo-englerian scenario [79]. Molecular phylogenetic studies based on a few DNA markers gave conflicting results: some claimed that gnetophytes are sister to conifers [35,39,80,81], some believed that the gnetophytes are sister to cupressophytes [40,41,82], while others considered that the gnetophytes are sister to the family Pinaceae [36,37,38,41,81]. Furthermore, some suggested that the gnetophytes are sister to the rest of gymnosperms [83], while others implied that the gnetophytes are sister to the remaining seed plant groups [43,81]. Recent phylogenomic studies have consistently shown that the gnetophytes are sister to the family Pinaceae [8,9]. As a result, the gnetophytes were classified in the Pinopsida as a separate subclass Gnetidae together with Pinidae and Cupressidae [10]. Within this phylogenomic context, the gnetophytes are derived conifers.

## 3. Character Evolution

Evolution of morphological characters is better understood within a phylogenetic context. According to the result of recent phylogenomics of gymnosperms, the evolution of some previously used taxonomic characters in gymnosperms should be reconsidered.

*Ginkgo* shows transitional characters between cycads and conifers. The vegetative anatomy and macromorphology of *Ginkgo* are similar to conifers, e.g., monopodial growth of trunk, pycnoxylic wood anatomy in the long shoots, but the reproductive anatomy resembles that of cycads, e.g., boat-shaped pollen, branched pollen tube, and the flagellate sperms (thus zoidogamy). Because *Ginkgo* is sister to cycads but not to conifers [7,8,9], the similarities between *Ginkgo* and conifers should be considered as superficial and not synapomorphic, while the similarities between *Ginkgo* and cycads should be considered as synapomorphic or plesiomorphic characters. First, the monopodial growth pattern is present in ‘coniferophytes s.l.’ (including *Ginkgo*, conifers, and gnetophytes) but lacking in cycads. It seems that the monopodial growth of tree trunk evolved independently in *Ginkgo* and coniferophytes. Second, wood anatomy was considered to be important in the taxonomy of gymnosperms, so the pycnoxylic wood was used to define a coniferophyte, while manoxylic wood defined the cycads [17]. The pycnoxylic wood of *Ginkgo* may have been derived separately from that of conifers. Third, the ovulate organ of *Ginkgo* is axillary to scale leaves of the short shoot and was considered to be homologous to the seed scale complexes of conifers, and the female short shoot is morphologically equivalent to a female cone of conifers [84,85]. According to recent phylogenomic results, the compound nature of the female short shoot in *Ginkgo* originated independently from that of conifers. Fourth, *Ginkgo* shares some similarities in reproductive anatomy with cycads, i.e., bilateral seeds with the seed coat differentiated into three layers (viz. sarcotesta, sclerotesta and endotesta), the branched pollen tubes and the flagellate sperms. These similarities have long been considered to be plesiomorphic, and inherited from their common ancestors in the Paleozoic. Considering that the Paleozoic gymnosperms possessed zoidogamy as well [11], we believe that it is better to consider the zoidogamy as plesiomorphic, but not synapomorphic characters. As for the boat-shaped pollen and the branched pollen tube, we may consider them as synapomorphic characters between cycads and *Ginkgo*.

**Figure 2:**
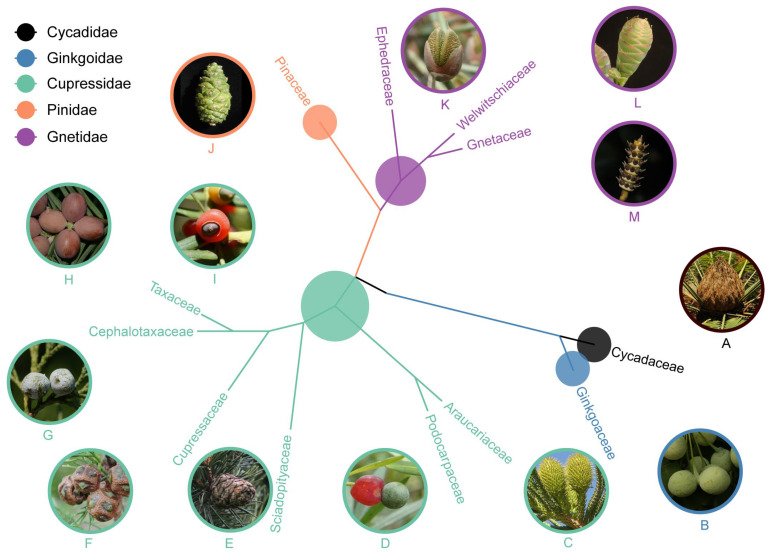
Female cone characters and phylogenomic relationships of extant families of gymnosperms. (**A**) *Cycas panzhihuaensis*; (**B**) *Ginkgo biloba*; (**C**) *Araucaria cunninghamii*; (**D**) *Podocarpus macrophyllus*; (**E**) *Sciadopitys verticillata*; (**F**) *Sequoiadendron giganteum*; (**G**) *Sabina chinensis*; (**H**) *Cephalotaxus sinensis*; (**I**) *Taxus cuspidata*; (**J**) *Pinus tabuliformis*; (**K**) *Ephedra rhytidosperma*; (**L**) *Welwitschia mirabilis*; (**M**) *Gnetum gnemon*.

Gymnosperms possess diverse female cones (Figure 2), and previous researchers laid much emphasis on female reproductive organs in their classification of gymnosperms. Sahni [86] classified gymnosperms into two groups, i.e., phyllosperms (cycads) and stachyosperms (*Ginkgo*, conifers and gnetophytes). The ovules are inserted on foliar organs in cycads, but on a modified shoot in *Ginkgo*, conifers and gnetophytes. Coulter and Chamberlain [30], Chamberlain [13], Bierhorst [69] and Fu and Yang [84] thought that the living gymnosperms consist of two major groups, i.e., cycadophytes and coniferophytes. Cycadophytes have simple female cones in which the foliar megasporophylls are spirally organized into a simple female cone. Coniferophytes include *Ginkgo*, conifers and gnetophytes and possess compound female cones with a number of morphological units on the cone axis (the seed scale complex consisting of the bract and its axillary secondary reproductive shoot).

The seed scale complex of conifers originated from a secondary reproductive shoot similar to the secondary shoot of Cordaitales and Voltziales by means of reduction and fusion of sterile and fertile foliar organs [84,87,88,89,90]. This kind of seed scale complex is typical of Araucariaceae, Pinaceae, Cupressaceae, Sciadopityaceae and Podocarpaceae. Researchers of the early 19th century [91] proposed this hypothesis based on tetratological evidence and was later upheld by multiple disciplinary evidence from vascular anatomy [5], ontogeny [92,93,94], paleobotany [87] and molecular data [95].

In the Taxaceae, however, there are no traces of such seed scale complexes; the seed is terminal to a twig and partially enclosed by a fleshy cupule from the fusion and modification of two foliar bracts (e.g., *Pseudotaxus* [96]). Florin [45,87] even considered that taxads evolved independently from other conifers and thus treated taxads as a separate class of Taxopsida. Herting and Stützel [97] disagreed with Florin’s model and thought that the seed scale complex is the funicular outgrowth of ovules.

Gnetophytes are derived conifers, the female cones are truly compound, and the chlamydosperms originated from the modification of secondary reproductive shoots with the fusion of a pair of bracteoles forming the envelope partially enclosing the inner seed [68]. However, it seemed that some early ephedroid plants possessed pedunculate chlamydosperms that were not organized into a compound cone (e.g., *Siphonospermum* [98]), and there are transitional forms between such simple pedicled chlamydosperms and the compact female cones of *Ephedra*, e.g., *Protognetella*, *Chengia* and *Liaoxia* [99,100,101]. If it is true, then the compound female cone of conifers and gnetophytes must have evolved independently. This is highly probable since some early fossil conifers bear simple female cones without seed scale complexes.

**Figure 3:**
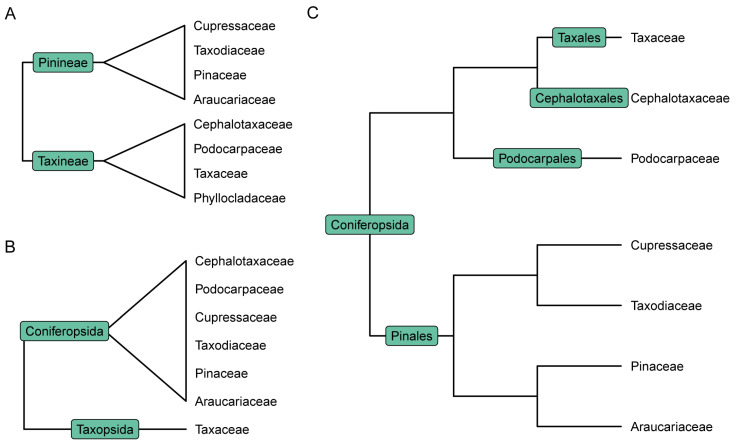
Historical classifications of conifers. (**A**) Keng [44]; (**B**) Florin [87]; (**C**) Cheng and Fu [46].

Based on the two types of female cones, conifers were divided in two groups: one having typical female cones that are woody and consist of multiple seed scale complexes, and the other possessing atypical female cones that are usually fleshy and consist of a few seed scale complexes or even only seeds and no seed scale complexes. Li [102], Keng [44] and Cheng and Fu [46] classified conifers using this character (Figure 3). However, there were different opinions on the evolutionary status of the two types of female cones, i.e., which type is more primitive in conifers. Many botanists in the 19th century classified *Ginkgo* in the taxads because of the superficial resemblance of the terminal ovulate organs [103]. People separated *Ginkgo* from the taxads and considered that cycads and *Ginkgo* are primitive gymnosperms soon after Japanese botanists discovered the flagellate sperms in *Ginkgo* and cycads [26,27,28]. These authors thought that the taxads have close relationships to *Ginkgo* and thus treated the reduced female cone type of the taxads as more primitive than the typical female cones [14,44,86,104,105,106,107,108,109,110]. Florin [45,87] established that the Taxaceae originated early in the Triassic and thought that the reduced female cone of the Taxaceae independently evolved from the reproductive shoot of *Lebachia*. For this reason, he separated the family as an independent class Taxopsida from the Coniferopsida (=Pinopsida). Miller [110] conducted a cladistic analysis based on female cones and found that the Taxaceae diverged from the Utrechtiaceae while other conifer families are linked to the Majoniaceae.

Some other authors, e.g., Chamberlain [13], Pilger and Melchior [31], Cheng and Fu [46] and Fu et al. [56], considered that the reduced atypical female cone type of taxads is derived. The Cephalotaxaceae bear typical female cones, though the seed possess a fleshy seed coat layer, which differs from the aril of the Taxaceae [58]. Within the recent phylogenomic context, the Taxaceae are sister to the Cephalotaxaceae with the Cupressaceae as the outgroup [8,9], suggesting that the reduced ovulate organs in the Taxaceae have been derived from typical female cones [89].

According to phylogenetic/phylogenomic studies, the Pinaceae and the gnetophytes form the gnepine clade, which is sister to the cupressophyte clade, and includes the rest of the conifer families (Figure 1E). This implies that the typical female cone is ancestral and the atypical female cones actually originated multiple times in the Podocarpaceae, the Cephalotaxaceae and the Taxaceae [90]. In the Cupressaceae, the female cones have also evolved towards a minimal and fleshy cone, e.g., some junipers bear fleshy cones with oligomerous seed scale complexes [111]. The independent evolution of reduced and fleshy cones in conifers may have been driven by animal dispersal of seeds [112].

## 4. Conclusions

Historically, the systematic position of a few problematic gymnosperm groups has been debated for a long time. Only the recent phylogenomic studies have given rise to consistent results. The living cycads contain two families, i.e., the Cycadaceae and the Zamiaceae. *Ginkgo* is sister to cycads. The conifers are paraphyletic with regard to gnetophytes, and the remaining conifer families (cupressophytes) comprise the core conifers; conifers and gnetophytes constitute the newly defined coniferophytes. *Sciadopitys* is classified in the Sciadopityaceae, while taxodiaceous genera should be included in the Cupressaceae. The family Cephalotaxaceae is distinct from the Taxaceae; gnetophytes constitute a monophyletic group that is sister to the family Pinaceae. Due to parallel evolution, organization and fleshiness, female cones should not be considered as important characters defining the clades above family level.

## Figures and Tables

**Table 1:** Summary of the gymnosperm classification of Yang et al. [10].

Class	Subclass	Order	Family
Cycadopsida	Cycadidae	Cycadales	1. Cycadaceae (1/126)
			2. Zamiaceae (9/255)
Ginkgoopsida	Ginkgoidae	Ginkgoales	3. Ginkgoaceae (1/1)
Pinopsida	Cupressidae	Araucariales	4. Araucariaceae (3/40)
			5. Podocarpaceae (20/181)
		Cupressales	6. Sciadopityaceae (1/1)
			7. Cupressaceae (31/169)
			8. Cephalotaxaceae (1/10)
			9. Taxaceae (5/29)
	Pinidae	Pinales	10. Pinaceae (11/272)
	Gnetidae	Ephedrales	11. Ephedraceae (1/70)
		Gnetales	12. Gnetaceae (1/46)
		Welwitschiales	13. Welwitschiaceae (1/1)

## Data Availability

All data are included in this paper.
